# Editorial Notes

**Published:** 1950-01

**Authors:** 


### Editorial Notes

**Devaluation** To a scientific association the fall in the value of money presents itself primarily as a rapid rise in expenses. Even those bodies whose membership is steadily increasing are compelled to raise their subscriptions.

| | 1948 | | | 1950 | | |
|---|---|---|---|---|---|---|
| | £ | s. | d. | £ | s. | d. |
| British Medical Association .. | 3 | 3 | 0 | 4 | 4 | 0 |
| Royal Society of Medicine .. | 4 | 4 | 0 | 6 | 6 | 0 |
| Medical Defence Union .. .. | 1 | 0 | 0 | 2 | 0 | 0 |

Our own Society has so far retained the rate fixed in 1919: but the balance sheets show that each year expenses exceed income and our reserves are melting away.

How seriously these changes affect the *Journal* our readers can see:

Cost of producing the July, 1949, issue at rates prevailing at different dates:

| | £ | s. | d. |
|---|---|---|---|
| 1938 .. .. .. | 65 | 11 | 0 |
| 1945 .. .. .. | 82 | 1 | 6 |
| 1948 .. .. .. | 97 | 8 | 6 |
| 1950 .. .. .. | 130 | 17 | 0 |

or about £5 10s. a page.

Cost of production has doubled in twelve years.* A larger circulation goes some way to meet the increase, but we have had to raise the subscription from half-a-guinea to sixteen shillings. We have adopted smaller type and cheaper paper: the 1950 cost for the same issue in the 1938 style would be £183. We have omitted lists of Past Presidents, subscribers, books received and many examination results.

* "In 1914 each number was produced for £36 ; in 1919 . . . £62." *Jl.*, 1919, 36, 177.

But the main economy forced upon us is a severe limitation of the length of original articles. And it is this which gives the most trouble. Oh for the days when we could print each article as it was received, and an obituary notice ran to five pages, with portrait! For when we " condense " an article the author naturally resents the sacrifice of his carefully chosen phrases—and it is even worse if we ask *him* to do it. In effect we find ourselves committed to quite considerable labour to make ourselves thoroughly unpopular!

We take this opportunity to apologize for the annoyance this curtailing must produce; to thank the authors for their patience and co-operation; and to express the hope that they will continue to support us by providing the material without which the *Journal* could not survive: adding the appeal traditionally exhibited in " Far West " bars, " Don't shoot the man at the piano—he is doing his best ".

